# Birth-Related Injuries Among Women: A Persistent Dilemma in Rural Uganda

**DOI:** 10.5334/aogh.5502

**Published:** 2026-11-12

**Authors:** Innocent Ayesiga, Darlson B. Kwikiriza, Primrose Magala, Enock Mukiibi, Lydia Demetriou, Denise Ead

**Affiliations:** 1 Department of Community Health, Mbarara University of Science and Technology, Mbarara, Uganda; 2 Maternal Health Department, Glory Divine Hospital, Ibanda, Uganda; 3 Department of Medical Retina, Moorfields Eye Hospital NHS Trust, London, United Kingdom; 4 Department of Research, Ubora Foundation Africa, Kampala, Uganda; 5 Reaching East Africa’s Children, Stockport, United Kingdom

**Keywords:** birth-related injuries, fistula, gynaecology, rural women, dignity, respectful maternity

## Abstract

There is a neglected burden of birth-related injuries among women in rural Uganda. Maternal morbidity deserves equal priority to maternal mortality. Preventable conditions including obstetric fistula, pelvic trauma, chronic pain, and psychological trauma persist because of health-system weaknesses and structural inequities. Strengthening emergency obstetric care, respectful maternity care, rehabilitation services, maternal morbidity surveillance, and broader social investments in education, poverty reduction, and women’s empowerment is essential to achieving safer and dignified motherhood.

## Key Message

Birth-related injuries among women continue to persist in rural Uganda and necessitate collective action from all stakeholders for better outcomes among the affected.

## Background

Maternal health conversations in sub-Saharan Africa often focus on mortality. Yet for every woman who dies during childbirth, many more survive with devastating and lifelong birth-related injuries, with approximately 475,000 women affected globally every year [1]. Maternal morbidity deserves equal attention to maternal mortality. Women in rural Africa often survive childbirth with disabilities that would have been entirely preventable elsewhere [2]. In rural Uganda, these injuries remain a neglected and under-prioritised public health crisis. This crisis is often hidden behind national statistics celebrating increased facility deliveries and declining maternal mortality ratios. Uganda is among the countries with the highest birth-related injuries globally, with an estimated prevalence of 19.2 per 1,000 women of reproductive age affected and over 2,000 cases reported annually [2]. Obstetric fistula, pelvic floor trauma, postpartum haemorrhage complications, uterine rupture, chronic perineal tears, neuropathies, infertility, and psychological trauma continue to shape the lives of thousands of women [3]. The persistence of these injuries reflects not only biomedical failures but also structural inequities such as poverty, lack of awareness among rural mothers about the risks, and long distances to health facilities [2].

## Risk Factors to Obstetric Injuries

Obstetric fistula remains one of the most severe and symbolic childbirth injuries in Uganda. It is often caused by prolonged obstructed labour without timely emergency obstetric intervention, particularly caesarean section [4]. Despite the actual statistics concerning the prevalence of obstetric fistulas being unknown, it is estimated that over 200,000 women are living with fistulas in Uganda, with an average annual incidence of 2,000 cases [3, 5]. A survey done between 2022 and 2023 among 24 healthcare facilities in the country found 1,129 women managed for obstetric fistula and other birth injuries. Nearly 4 in 10 were found to have sustained birth injuries during their first pregnancy [3]. Women affected by fistula experience continuous leakage of urine or faeces, chronic infections, infertility, social isolation, stigma, marital breakdown, depression, and economic abandonment. The persistence of birth-related injuries in rural Uganda is inseparable from poverty. Women living in remote districts frequently face long distances to health facilities, inadequate transportation, and poorly equipped maternity units [4]. Labour may begin at home, and delays in recognising complications, seeking care, reaching facilities, and receiving appropriate treatment often become fatal or disabling. These ‘delays’ have long been described in maternal health literature, but evidence from Uganda has further described a ‘fourth delay’: neglect and poor responsiveness within health systems themselves [4]. For example, only 26% of the 1,129 women who had obtained treatment from 24 health facilities in the 2022/2023 Financial Year survey received treatment within the year of the injury [6]. This clearly demonstrates the delay these women experienced in seeking necessary care after the injury. Another qualitative analysis from Soroti, Uganda, demonstrated that women spent between months and 21 years to acquire fistula treatment [2]. This delay was also attributed to limited understanding of the condition’s dynamics, especially due to inaccurate and limited information.

Additionally, the burden of maternal injuries in Uganda is socioeconomically patterned [2]. Rural girls are more likely to marry early, experience adolescent pregnancies, and have limited access to reproductive health education. Women with low educational attainment and limited financial autonomy are less able to seek timely obstetric care. For example, Bomboka et al. found that 43% of the 149 fistula survivors had only primary education, with 73.1% below 24 years [7]. Malnutrition during childhood contributes to stunted pelvic development, increasing the risk of obstructed labour [8]. These overlapping vulnerabilities create a cycle in which childbirth becomes a dangerous event rather than a safe reproductive experience. Although Uganda has increased skilled birth attendance, the quality of maternity care remains inconsistent. Rural health centres frequently experience shortages of trained personnel, blood supplies, surgical capacity, anaesthetic services, and referral systems [9]. In many facilities, overworked midwives and clinicians manage overwhelming patient volumes with inadequate resources. The influence of traditional birth attendants has also remained prevalent in rural areas, and often, when complications arise, there are delays in referral to an appropriate health facility [10]. Thus, preventable complications such as obstructed labour, severe postpartum haemorrhage, and birth trauma continue to occur.

## Psychological Impact and Health System Linkage

Beyond physical injuries, the psychological consequences of traumatic childbirth are rarely acknowledged in maternal health programming. Women who experience prolonged labour, stillbirths, fistula, emergency surgery, or disrespectful maternity care may develop depression, anxiety, post-traumatic stress symptoms, and social withdrawal [6]. Yet mental health screening and psychosocial support remain largely absent from postnatal services in rural Uganda [11]. More than one-third of women in low- and middle-income countries reported mistreatment during childbirth, including verbal abuse, lack of consent, neglect, and physical mistreatment [12]. Such experiences discourage future facility-based deliveries and erode trust in health systems. In many rural Ugandan settings, women continue to report harsh language, abandonment during labour, and poor communication from healthcare providers [1].

Birth-related injuries should not be viewed solely as clinical conditions. They are markers of health-system inequity and social injustice. The persistence of obstetric fistula in Uganda decades after its elimination in high-income countries reflects unequal access to timely emergency obstetric care. The consequences extend far beyond the individual woman. Families lose income when affected women can no longer work. Children may experience neglect when mothers are chronically ill or psychologically traumatised. Communities perpetuate stigma, especially towards women with fistula or infertility [11]. Marriages frequently collapse under the strain of chronic incontinence and social embarrassment [13]. Thus, maternal injuries create intergenerational cycles of poverty and exclusion.

## Call to Action

Addressing birth-related injuries in Uganda requires moving beyond narrow mortality-centred indicators. Maternal morbidity surveillance systems remain weak, and many childbirth injuries are underreported. Women delivering outside health facilities may never be captured in official statistics. A robust surveillance system must also capture the full burden of maternal injuries, for example, where and why they occur. This will enable targeted prevention rather than retrospective, reactive responses. Investing in community-level reporting and case definitions that include non-fatal injuries is essential to making these women visible in national data. Furthermore, postnatal follow-up services are inadequate, meaning injuries such as pelvic organ prolapse, chronic perineal tears, and urinary incontinence often go undiagnosed for years. Investment in comprehensive emergency obstetric and newborn care is essential, and Uganda has made significant progress, especially through regular audits and continuous professional development. However, rural health facilities still need reliable surgical theatres, blood transfusion services, ambulance systems, specialist-trained obstetric staff, and functioning referral pathways. Community health education is required and must emphasise early recognition of labour complications and birth preparedness. Equally important is strengthening respectful maternity care through provider training, accountability systems, and patient-centred approaches.

Uganda must also prioritise rehabilitation and reintegration services for women already living with birth-related injuries. Surgical repair camps for obstetric fistula have provided relief for many women, but access remains limited and inconsistent [14–16]. Community reintegration programmes can help reduce stigma and restore dignity. Research institutions and policymakers should increasingly focus on maternal morbidity as a distinct research agenda. Greater investment is needed in epidemiological studies, implementation research, and health-system interventions to prevent maternal injury in rural Uganda [16]. Girls’ education, delayed marriage, poverty reduction, nutrition improvement, road infrastructure, and women’s empowerment are all maternal health interventions. Obstetric injuries cannot be eliminated through hospital-based strategies alone. They emerge from structural inequalities that begin long before labour starts.

Therefore, international partners, donors, and governments must resist framing maternal health success exclusively in terms of mortality reduction. Survival alone is not enough. A woman who survives childbirth with lifelong incontinence, chronic pain, infertility, or severe psychological trauma has not experienced safe motherhood in its fullest sense. The World Health Organization (WHO) recognises that pregnancy-related complications can leave women with lasting disabilities [3]. These can include chronic pain, urinary incontinence, shame, and psychological distress. Preventing fistula occurrence through quality obstetric care is therefore fundamental to improving overall outcomes [17]. Timely, quality obstetric care can enhance women’s dignity.

## Data Availability

No data were generated while developing this manuscript.

## Patient and Public Involvement

Patients or the public were not involved in the design, conduct, reporting, or dissemination plans of our research.
